# Advances in mycobacteriophage research: from lytic mechanisms to one health applications

**DOI:** 10.3389/fcimb.2026.1856292

**Published:** 2026-06-16

**Authors:** Ruyi Han, Lingjun Shen, Victoria Chen, Xingyan Tan, Ni An, Donghui Ke, Yuqing Li, Jumei Zeng

**Affiliations:** 1 West China School of Public Health and West China Fourth Hospital, Sichuan University, Chengdu, China; 2 State Key Laboratory of Oral Diseases, West China Hospital of Stomatology, Sichuan University, Chengdu, China

**Keywords:** antimicrobial resistance, lytic mechanisms, mycobacteriophages, one health, phage therapy, phage-host interaction

## Abstract

Mycobacteriophages are viruses that specifically infect mycobacterial hosts. Owing to their ability to penetrate the unique and complex cell wall barrier of mycobacteria, mycobacteriophages have recently emerged as promising tools for the diagnosis and treatment of drug-resistant mycobacterial infections. This review systematically summarizes the classification, genomic features, lytic and lysogenic mechanisms of mycobacteriophages, as well as the intricate defense and counter - defense strategies between phages and their hosts. Furthermore, from a One Health perspective, we explore the diverse applications of mycobacteriophages across human health, animal health, and environmental management, including rapid detection, treatment of drug-resistant infections, environmental surveillance and occupational exposure management. Collectively, mycobacteriophages are promising for combating drug-resistant infections and playing an important role in integrated One Health strategies.

## 1 Mycobacteriophages

Mycobacteria are a group of bacteria characterized by a sophisticated cell wall rich in mycolic acids, glycolipids, and glycopeptidolipids, which confer intrinsic resistance to multiple antibiotics as well as acid-fast staining property. This genus primarily includes the *Mycobacterium tuberculosis complex* (MTBC), *Mycobacterium leprae*, and *nontuberculous mycobacteria* (NTM). NTM are classified into rapidly growing mycobacteria (RGM) and slowly growing mycobacteria (SGM) (Maleki and Moaddab, 2025; Wetzstein et al., 2024), Notably, *M. abscessus*, *M. chelonae*, *M. fortuitum*, and *M. smegmatis* belong to the RGM group (Maleki and Moaddab, 2025). Conversely, the *M. avium complex*, *M. kansasii*, and *M. ulcerans* are categorized as SGM (Matos et al., 2025).

Mycobacteriophages specifically infect the genus Mycobacterium, all of them are double-stranded DNA (dsDNA) phages belonging to the order Caudoviricetes (tailed phages). Mycobacteriophages can penetrate the exceptionally thick, hydrophobic waxy cell wall unique to mycobacteria and establish productive infection, a feature that underpins their application in diagnosing and treating infections caused by pathogenic mycobacterial. High-throughput sequencing and systems-level bioinformatics increasing the identification and annotation of bacteriophages, 14,956 mycobacteriophages have been identified and characterized (as of 2026; PhagesDB), making them one of the most genomically diverse phage lineages described to date. These extensive genomic resources offer unprecedented opportunities for deciphering the mechanisms of phage-host interactions and developing innovative anti- mycobacterial strategies (Bonacorsi et al., 2024).

### 1.1 Morphology and classification

Most mycobacteriophages identified to date have been isolated on *M. smegmatis mc 2155* as the primary propagation host. All discovered mycobacteriophages are classified belong to the class *Caudoviricetes* (which encompasses the former order Caudovirales) and exclusively possess double-stranded DNA (dsDNA) genomes. Based on differences in their tail structure, mycobacteriophages are further subdivided into three morphological families: contractile-tailed, long non-contractile-tailed, and short-tailed—historically referred to as the families *Myoviridae*, *Siphoviridae*, and *Podoviridae*, respectively (Hatfull, 2018). Members of the Contractile-tailed phages have been identified exclusively within cluster C (Hatfull et al., 2010). By comparison, phages with elongated, non-contractile tails—historically classified as Siphoviridae—distinguished by their elongated, non-contractile tails, constitute the predominant morphological group, accounting for over 90% of all identified mycobacteriophages (Pope et al., 2011). Newly identified phages such as *Lang* (subcluster G1) and *CRB2* (subcluster B9), display a typical “tadpole” morphology with subcluster-specific variations in head-to-tail proportions (Cresawn et al., 2015; Lang et al., 2023). Conversely, short-tailed phages remain exceptionally scarce among mycobacteriophages, with merely sporadic documentation (Pope et al., 2011; Sarkis and Hatfull, 1998). Regarding capsid architecture, most mycobacteriophages display isometric icosahedral heads with diameters ranging from 40~80 nm. A minority, such as the *Catdawg* phage, exhibit prolate heads (spindle-shaped) characterized by axial ratios of approximately 2.5:1 to 4:1 (Hatfull et al., 2010).

Concomitant with their striking morphological diversity, mycobacteriophages display remarkable genetic heterogeneity at the genomic level, exemplifying characteristic mosaic evolution wherein phage genomes are composed of functionally distinct modules derived from varied ancestral sources (Hatfull, 2015). Research field has widely embraced a three-tier “Cluster-Subcluster-Singleton” classification framework grounded in genomic sequence similarity and phylogenetic clustering (Hatfull, 2025). Under this taxonomic system, reported mycobacteriophages are currently assigned to at least 29 defined clusters and over 10 singletons, with each cluster further subdivided into multiple subclusters (O'Connell et al., 2023). This hierarchical taxonomy not only underscores the extensive evolutionary divergence among these phages, but also implies substantial variation in infection strategies, life cycles modalities, and host adaptation mechanisms (Dedrick et al., 2017a). In current study, we summarized the key characteristics of major mycobacteriophage clusters and presented representative phages within each cluster (**Table 1**).

**Table 1:** Comparative characteristics of major mycobacteriophage clusters.

Cluster	Subcluster	Average genome size(bp)	Average GC content(%)	Representative phage	Key characteristics
A	A1-A14, A16- A22	51551	63.3	L5, D29, SWU1	Largest cluster; most subclusters infect M. tuberculosis. Notably, subcluster A15 is restricted to Gordonia spp.
B	B1-B13	68884	67.1	Hedgerow, Rosebush	Lacks integrase genes; some members rely on the host Wag31 protein to establish infection.
C	C1-C2	155619	64.6	Rizal	The only cluster exhibiting contractile-tailed morphology with contractile tails; possesses the largest genome sizes and infects only rapidly growing mycobacteria.
D	D1-D2	64791	59.5	Chill, PLot	Typically exhibits a lytic lifecycle; experimental evidence indicates that stable lysogens cannot be established.
E	–	75428	63.0	ABCat, Douge	The largest cluster without defined subclusters; genomes display a unique modular organization.
F	F1-F7	57368	61.4	Tweety, Fruitloop, Squirty	Exhibits high gene content flux (HGCF); Tweety harbors anti-defense genes with tetratricopeptide repeats (TPR).
G	G1-G5	42386	66.9	Lang, Grizzly, Sweets, BPs	Subcluster G1 members share >95% genomic similarity; features integration-dependent immunity (attP within the repressor).
H	H1-H2	69128	57.3	Damien, Oaker, Konstantine	Contains orphan genes with unknown functions.
I	I1-I2	50700	66.4	Brujita, Che9c	Infects only Mycobacterium smegmatis and lacks lysogenic capability.
J	–	110897	60.8	Omega, Minerva	Contains IS110-like transposons and introns; Marvin exhibits anomalous tail protein gene localization at the right end.
K	K1-K8	59970	66.8	Adephagia, TM4	Features a highly conserved 13-bp repeat sequence; broad host range including M. tuberculosis.
L	L1-L5	74570	58.8	Archie, Rose5	Lower GC content; some members are capable of infecting M. avium.
M	M1-M3	81571	61.3	Auspice, Bongo	Siphoviridae with exceptionally long tails; encodes a high density of tRNA genes.
N	–	42931	66.2	Xeno, Snekmaggedon	Encodes multi-subunit restriction systems to defend against heterologous phage infection.
O	–	71102	65.3	Winget, Zakhe101	Some members harbor mycobacterial polymorphic mobile elements (MPME).
P	P1-P6	47820	67	Arib1, Tortellini, Phayonce, Xavia	Subcluster P1 is restricted to Mycobacterium; P2-P6 exhibit cross- genus infection (Gordonia, Corynebacterium).
Q	–	53917	67.5	Giles	The lysin B gene is non-essential; its deletion results in reduced plaque size.
R	–	71339	56	Nilo, Zenon	One of the clusters with the lowest GC content.
S	–	64976	63.3	Beelzebub, Gattaca	Features a highly compact arrangement of structural genes.
T	–	42724	66.2	Bryson, Nette	Possesses a relatively small genome containing a core lysis gene module.
U	–	67817	50.3	Patience	Represents the cluster with the lowest GC content; codon usage bias differs markedly from that of the host.
V	–	77907	56.9	Cosmo	Contains unique tail assembly genes.
W	–	61013	67.5	Jeon	Some members encode HNH endonucleases.
X	–	88036	56.6	Gaia	Singleton cluster; genome contains unique regulatory genes.
Y	–	76506	66.4	Faiyaz	Singleton cluster; encodes integrase and attP site.
Z	–	50806	66.9	32HC, Rem711	Two-member cluster; structural gene organization shows partial homology with cluster A.
AB	–	49213	58.7	Muddy, Salvus	Two-member cluster; characterized by relatively low GC content.
AC	–	70028	49.8	Cuke	Singleton cluster; among those with the lowest GC content.
Singleton	–	62132	64.1	DS6A, Kumao, MooMoo	Lacks closely related homologous genomes; DS6A is capable of directly infecting M. tuberculosis.

As of March 2026, comprehensive genome sequencing and functional annotation have been completed for over 2,600 mycobacteriophages. Their genome sizes vary from ~40 kb to > 160 kb, further highlighting the profound diversity in genetic architecture and functional capacity characteristic of mycobacteriophages (Hatfull, 2018; Pedulla et al., 2003).

### 1.2 Genomic characteristics and conserved gene-encoded proteins

All mycobacteriophage genomes are composed of double-stranded DNA (dsDNA) and exist in either linear or circular conformations. While most mycobacteriophages including *Chy1*, *Chy2*, *Chy4*, and *Chy5* exhibit canonical linear genome architectures, certain isolates initially assemble as circular contigs. Restriction endonuclease mapping has subsequently confirmed that such genomes are fundamentally linear with circularity representing an assembly artifact (Das et al., 2024). Mycobacteriophage genome sizes display substantial heterogeneity, ranging from 38,120 bp to 164,602 bp, with the majority of phages clustering within the 40–60 kb interval (Pope et al., 2015; Tan et al., 2025). The gene content typically comprises 58–88 coding sequences, with an overall gene density of approximately 0.91-0.97, indicating a compact nature of these genome. Analysis of the guanine-cytosine (GC) content of mycobacteriophage genomes indicates the values between 63.68% and 66.86%, closely paralleling the 57%-65.6% GC range observed in their mycobacterial hosts (Pope et al., 2015). The convergence in base composition strongly suggests extensive co-evolutionary adaptation between mycobacteriophages and their hosts during prolonged coexistence.

Mycobacteriophage genomes are characterized by extensive modularity (Hatfull et al., 2010), in which essential functional genes are organized in discrete clusters, while non-structural genes are predominantly confined to the right genomic arm, reflecting well-defined functional compartmentalization (Pedulla et al., 2003). However, structural genes of phage *Chy3* flanking the integrase gene bilaterally, in contrast sharply with the unilateral distribution observed in typical mycobacteriophages. Structural genes of mycobacteriophages primarily encode components of the capsid, tail, and associated structural proteins that are essential for accurate virion morphogenesis, as well as host recognition and productive infection. Conserved head- associated proteins include the major capsid protein, scaffolding protein, and portal protein, each governing distinct processes of capsid morphogenesis, assembly regulation, and DNA packaging, respectively. Tail-associated conserved proteins encompass the major tail protein, minor tail proteins, tape measure protein (TMP), and tail fiber-related proteins. Notably, TMP is typically the longest conserved gene in the phage genome, and its length is directly correlated with tail length (Taylor et al., 2018). A highly conserved motif 3 is universally present in TMPs, this motif is implicated in peptidoglycan hydrolase activity and potentially contributes to host cell wall penetration (Piuri and Hatfull, 2006; Sun et al., 2014). Tail fiber proteins mediate bacteriophage host recognition and attachment, thereby determining phage specificity for host and serving as the critical molecular determinants of infection specificity and lytic efficacy.

The lytic system of mycobacteriophages comprise a canonical two-component mechanism encoded by lysin (*lysA* and *lysB*) and holin genes. LysA primarily catalyzes hydrolysis of the host peptidoglycan backbone, whereas LysB specifically targets lipid components such as mycolic acids (Gigante et al., 2017), this concerted activity ensures comprehensive mycobacterial cell wall degradation (Payne and Hatfull, 2012). Notably, the LysA, LysB, and holin genes of*Chy1*, *Chy4*, and *Chy5* exhibit 99%-100% sequence identity to their counterparts in phage *D29*, with the holin sequences being completely identical (Gan et al., 2016). Holins are hydrophobic membrane proteins that form pores in the host cytoplasmic membrane to facilitate lysin egress, and their regulated expression plays a critical role in governing the timing and efficiency of the lytic cycle. Alternatively, holin-deficient mycobacteriophages employ signal peptide-mediated secretion pathways for lysin export, enabling autonomous host cell lysis (Payne and Hatfull, 2012).

The integration system of lysogenic mycobacteriophages comprises genes encoding integrase (Int), phage attachment site (attP), and the repressor. Integrase constitutes a cluster-spanning conserved core, predominantly representing tyrosinerecombinase (e.g., phages *L5*, *Bxb1*, *Ms6*, and *Che12*) with sporadic serine recombinases (e.g., *φRv1*, *φRv2*, and certain phages in clusters C and I). These enzymes catalyze site-specific recombination between the phage attP and the bacterial attB loci (Van Duyne, 2007), a process fidelity relies upon the 43 bp core sequence homology between attP and attB. The repressor proteins maintain lysogeny through binding phage genomic regulatory elements and transcriptionally repressing the expression of lytic genes. For instance, the repressor of phage *Chy1* exhibits complete sequence identity to p72 gene of phage *D29*, yet *D29* harbors an N-terminal deletion abrogating helix- turn-helix (HTH) motif formation, thereby conferring obligately lytic behavior (Dedrick et al., 2017b). Conversely, *Chy2* repressor is identical to the gp33 of phage *Halo*, ensuring robust lysogenic stability (Brown et al., 1997). Furthermore, mycobacteriophages encode excisionase (Xis)can trigger prophage excision and lytic cycle induction (Dedrick et al., 2017a).

Beyond core functional genes, mycobacteriophages possess conserved genetic determinants for DNA replication, repair, and metabolic processes, encompassing DNA polymerase, ribonucleotide reductase, and thymidylate synthase. These gene products constitute autonomous nucleic acid metabolic machineries, with DNA polymerases safeguarding replicative fidelity. DNA polymerase III subunit genes are detectable in select large-genome mycobacteriophages, particularly Cluster A representatives such as *L5*. Ribonucleotide reductase catalyzes deoxyribonucleotide biosynthesis, furnishing essential precursors for genome replication (Hatfull, 2012). Several conserved loci also encode transcriptional regulatory proteins that modulate phage-host interactions. For instance, the GL33 genes of phages *Chy1* and *Chy4* encodes a MerR superfamily regulators implicated in host oxidative stress and antibiotic exposure modulation. Additionally, the pfam05305 family members are highly conserved across *M. tuberculosis* and *M. avium* (Jacobs-Sera et al., 2012). Although their precise functions remain to be fully elucidated, they are hypothesized to contribute to phage-host adaptation. Significantly, mycobacteriophage genomes encode abundant hypothetical proteins- genes of unknown or merely predicted functions. This feature highlights the considerable, yet largely unexplored, functional potential of mycobacteriophages (Sullivan et al., 2021).

### 1.3 The lysogenic and lytic cycles

Consistent with other bacteriophages, the life cycle of mycobacteriophages conform to canonical virus-host co-evolutionary principles, fundamentally bifurcating into the lytic cycle and the lysogenic cycle. Upon infection, lytic phages maintain extrachromosomal genome replication, eventually culminating in host cell lysis and progeny release. In contrast, temperate phages integrate as prophages into the host chromosome, establishing heritable lysogenic state that persist through host cell division, with bidirectional cycle switching responsive to environmental cues (Hatfull, 2018). Genomic and phenotypic analyses of >2,600 sequenced mycobacteriophages indicate that ~58% are temperate phages (lysogenic-competent) and ~42% are virulent (obligately lytic). Temperate phages predominantly affiliate within Clusters A, G, and K, and their genomes encode key regulatory genes such as integrase (Int), repressor. Conversely, lytic phages cluster within Clusters B, C, and O, characterized by absence of integration-related elements and retention of lysis-exclusive gene sets (Bonacorsi et al., 2024). The lytic or temperate status correlates significantly with host pathogenicity. About 65% of temperate phages can infect *M. tuberculosis*, while *M. abscessus*-infecting phages (though less numerous), exhibit increased lytic prevalence and markedly restricted host specificity (Wetzel et al., 2023).

**Figure 1:**
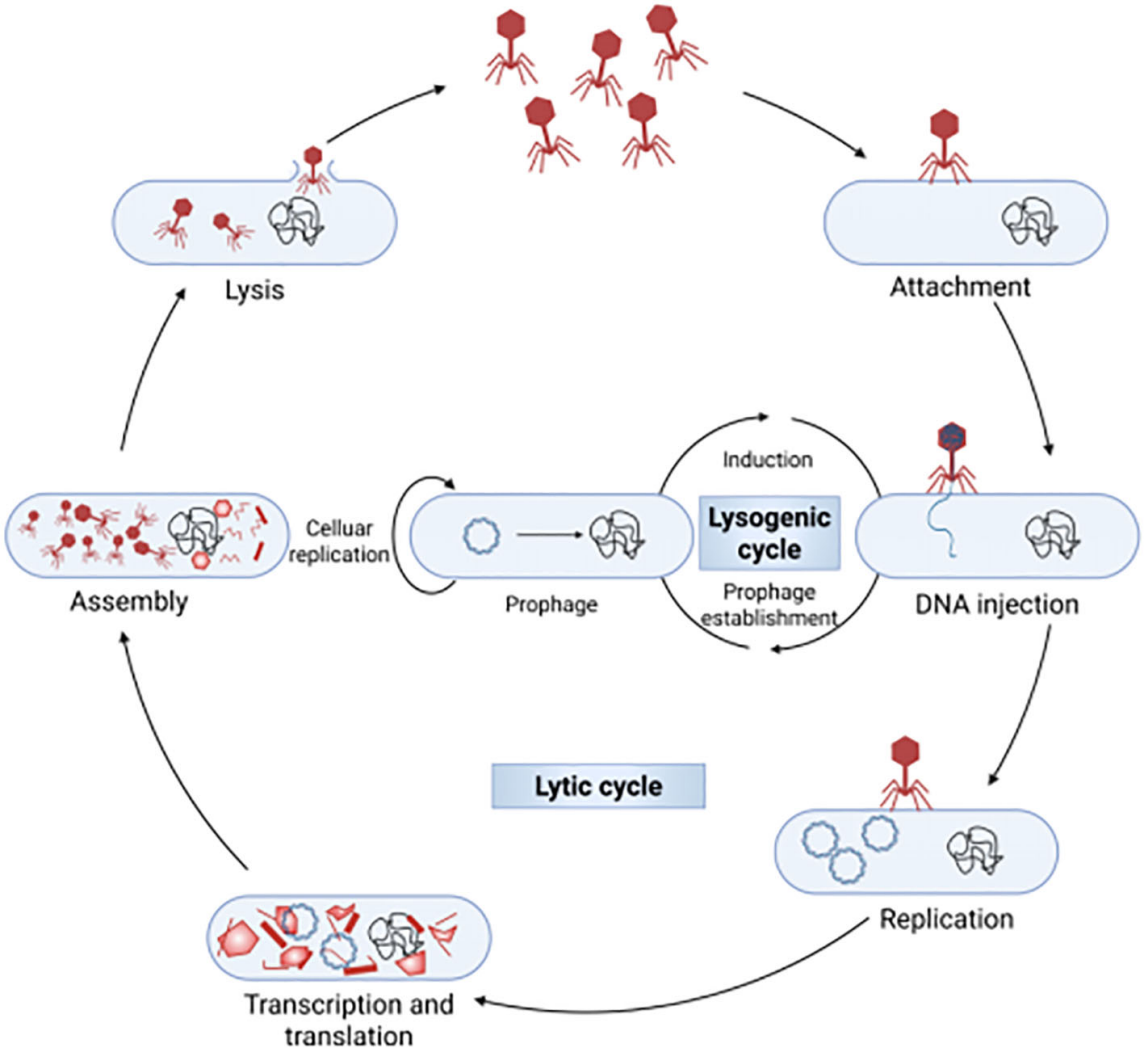
Lytic and lysogenic cycles of mycobacteriophages. (Created in http://BioRender.com).

The lytic cycle serves as the fundamental pathway for rapid phage propagation and dissemination, encompassing five consecutive phases: attachment, penetration, biosynthesis, assembly, and host lysis (**Figure 1**). Each step governed by intricate regulatory networks and orchestrated structural proteins interactions. Infection commences via specific engagement between phage tail-associated proteins and cognate host receptors. The distinctive cell envelope of mycobacteria-composed of mycolic acids, peptidoglycan, and arabinogalactan-dictates receptor diversity. Identified receptors include glycopeptidolipids (GPLs), trehalose polyphleates (TPPs), and mycolic acid derivatives. For instance, cluster K phages utilize tail fiber proteins for targeted adsorption to *M. abscessus* TPPs, whereas cluster A phages initiate attaching by recognizing specific glycosylated epitopes on mycolic acids (Wetzel et al., 2023). Post-attachment triggers phage tail conformational transitions, enabling the hydrophobic domains of the tape measure protein (TMP) to traverse the tail tube and penetrate the multilayered cell wall architecture. Notably, the TMPs of certain virulent phages also exhibit ancillary peptidoglycanase activity, facilitating genome translocation across the cell wall barrier. The conserved MSTF motif within TMP binds and locally hydrolyzes peptidoglycan, thereby forming a 2–3 nm conduit for double- stranded DNA genome delivery into the host cytoplasm (Ge and Wang, 2024).

Following cytoplasmic entry, mycobacteriophage gene expression proceeds in a strictly orchestrated temporal regulation. Representative phages such as *D29*, *L5*, and *Bxb1* exhibit a characteristic transcriptional cascade in which early genes are preferentially expressed immediately after infection. These genes primarily encode proteins involved in DNA replication and transcriptional regulation, such as DNA polymerase and transcriptional regulators, enabling the phage to commandeer host nucleotide acid metabolism for phage genome amplication (Hatfull, 2018). As replication progresses, the phage transitions into the middle expression phase, yielding major capsid proteins, tail scaffold components, and auxiliary assembly chaperones. Late-phase transcription subsequently deploys lysis cassettes to prepare for ultimate host disruption (Hatfull and Hendrix, 2011). During the late phase of infection, progeny virion morphogenesis follows a rigorous modular assembly principle, portal-mediated genome translocation drives packaging of replicated DNA into procapsids constructed from major capsid proteins tail virions, followed by tail polymerization and accessory structure attachment to generate infectious progeny virions (Rao et al., 2023). Notably, intra-cluster conservation exceeding 90% amino acid identity among major capsid proteins, portal proteins, and tail assembly proteins underpins the molecular precision and structural fidelity characteristic of mycobacteriophage virion morphogenesis (Dedrick et al., 2017a).

Mycobacteriophage lysis operates through a distinctive tripartite system involving Lysin A, Lysin B, and Holin, coordinately targeting and efficiently degrading the architecturally complex mycobacterial cell envelope. Holins are small integral membrane proteins that aggregate in the host cytoplasmic membrane during the late phase of infection, where they form micron pores that facilitate the egress of endolysins. Lysin A executes peptidoglycan degradation via glycosidase or amidase cleavage of β- 1,4-glycosidic or amide bonds, Lysin B, a mycolylarabinogalactan esterase, specifically severs covalent mycolate-arabinogalactan bonds to the outer lipid while Lysin B, acts as a mycolylarabinogalactan esterase, specifically hydrolyzing the covalent linkage between mycolic acids and the arabinogalactan to compromise the thick lipid-rich outer barrier (Nair and Jain, 2024). Intriguingly, holin-deficient mycobacteriophages retain lytic competence through Sec- dependent secretion of N-terminal signal peptide- bearing Lysin A. Lysis kinetics typically spans 60–90 minutes, and under optimal multiplicity of infection (MOI) conditions, with single-cell burst sizes approximating 100 progeny virions (Harman-McKenna and De Buck, 2023). This has been demonstrated in one-step growth curve analysis with phage *KVT1* and is corroborated by D29 life cycle metrics.

Lysogeny constitutes an evolutionary adaptation enabling prolonged temperate phages-host coexistence, proceeding through three operationally defined phases including integration, maintenance, and induction, which was primarily mediated by precise regulatory control of integrase, repressor and ancillary factors (Hatfull, 2018). Temperate phages integrate their genomes into the host chromosome via site-specific recombination catalyzed by integrase (Int). Most mycobacteriophages preferentially target host tRNA loci (attB sites) to attenuate fitness costs. For example, phage *L5* integrates into the tRNA-Gly gene of *M. smegmatis*, and the disrupted host tRNA gene function restored through subsequent special sequence repair (Peña et al., 1996). In addition, the high-fidelity integration is further ensured by substantial sequence identity (up to 86%) between the phage attP site and conserved host chromosomal attB sequences. Mycobacteriophages have evolved additionally diversified non-canoical lysogenic strategies. Research on Cluster A phages has revealed that nine Cluster A prophages (including *Bxb1* and *L5*) map to six distinct attB sites (attB-1, attB-2, attB-8, attB-12, attB-13, attB-14) and deploy integration- dependent immunity. This unconventional architecture nests attP within the repressor coding sequence, generating C-terminally distinct phage-encoded versus prophage- encoded gene products. The phage-encoded repressor typically carries a C-terminal SsrA-like degradation tag, rendering it unstable and incapable of conferring immunity, mandating integration- dependent tag removal for functional repressor maturation, thereby establishing stable lysogeny and concomitant superinfection immunity (Dedrick et al., 2021, 2017). Furthermore, certain phages (e.g., cluster K, F, and some A) utilize a tRNA-dependent lysogenic system alternatively (Hatfull, 2014), where attB localization within host tRNA gene enables functional compensation for integration-disrupted host loci, thereby safeguarding lysogen viability. Generally, integrated prophages constitute 3%-8% of the host chromosome content without deleterious impact on core host metabolic (Canchaya et al., 2003).

The long-term stability of lysogenic state necessitates persistent repressor activity and rigorous suppression of lysis-associated gene transcription. Repressor-mediated occupancy of prophage operator or promoter regions enforces epigenetic silencing of the phage genome. Superinfection immunity is a critical defensive trait conferred upon lysogenic hosts. In this mechanism, the repressor dual-function to suppress resident prophage lytic genes and antagonize early gene expression in the invading phage through competitive promoter binding, thus protecting the host from secondary infection. The gp45-encoded repressor of phage *ZoeJ* confers cross- protective immunity against cluster K phages and significantly attenuates superinfection frequency (Dedrick et al., 2017a; Taylor et al., 2025).

The transition from lysogenic to lytic is primarily triggered by host stress signals, with the central switching mechanism of derepression via repressor proteolysis. Beyond canonical repressor control, some mycobacteriophages encode additional regulatory elements implicated in host stress circuitry. For instance, the gp29 of the cluster P phage *Phrann* encodes a predicted (p)ppGpp synthetase homologous to RelA. The lysogenic expression of gp29 potentially modulates host stringent response dynamics to facilitate adaptive prophage-host interplay, implicating the (p)ppGpp signaling pathway may play a previously underappreciated role in phage life cycle governance (Dedrick et al., 2017a). Moreover, spontaneously lytic induction and viral particles release from *L5* prophage in clinical isolates under *in vitro* conditions further suggests the existence of environmental triggers that remain to be fully elucidated.

### 1.4 Promising mycobacteriophages

The application of mycobacteriophages as genetic engineering platforms has undergone substantial expansion. The replicon system derived from phage *1S1* has been widely utilized to develop cross-species large-fragment cloning methodologies, enabling the successful cloning of 34 kb defense island fragments and effectively overcoming a critical technical constraints in mycobacterial large-fragment genomic manipulation (Wang et al., 2025a). In recent years, research on mycobacteriophages has achieved significant advances across multiple dimensions, particularly in elucidating phage-host interactions, dissecting underlying molecular mechanisms, and exploring their potential clinical application, thereby contributing to the establishment of a more comprehensive theoretical framework. **Table 2** summarizes mycobacteriophage candidates with potential applications in genome engineering, multifunctional exploitation of encoded products, and antimicrobial resistance mitigation.

**Table 2:** Descriptive comparison of promising mycobacteriophages.

Mycobacterio phage	Family	Cluster/sub cluster	Origin	Infect	Life cycle	Sequenced
D29	Siphoviridae	A/A2	Soil	M. smegmatis, M. tuberculosis, M. avium	Temperate	Yes
L5	Siphoviridae	A/A2	Soil/Lysogen	M. smegmatis, M. tuberculosis	Temperate	Yes
TM4	Siphoviridae	K/K2	–	M.smegmatis, M. tuberculosis, M. avium, MAP	Temperate	Yes
BPs	Siphoviridae	G/G1	–	M. smegmatis, M. tuberculosis	Temperate	Yes
Muddy	Siphoviridae	AB	Soil	M. smegmatis, M. abscessus M. smegmatis, M. tuberculosis,	Lytic	Yes
ZoeJ	Siphoviridae	K/K2	Soil	M. avium, M. bovis	Temperate	Yes
SWU1	Siphoviridae	A2	Soil	M. smegmatis, M. tuberculosis	Temperate	Yes
Leo	Siphoviridae	G-Like	–	M. smegmatis, M. tuberculosis	Lytic	No
DS6A	Siphoviridae	Singleton	–	M. tuberculosis complex (MTBC)	Temperate	Yes
Araucaria	Siphoviridae	Dori-like	Respiratory specimens	M.abscessus, subsp. bolletii	Temperate	Yes
Bxz1	Myoviridae	C	Soil	M. smegmatis, M. vaccae	Lytic	Yes
vB_MapS_FF 47	Siphoviridae	–	Cow manure	M. smegmatis, MAP	Lytic	Yes
Cjw1	Siphoviridae	E	–	M. smegmatis	Temperate	Yes
PDPRv	Siphoviridae	B/B1	Soil	M. smegmatis, M. tuberculosis	Lytic	No
Angel	Siphoviridae	G	Soil	M. smegmatis, M. tuberculosis	Temperate	Yes
FRAT1	–	–	–	M. smegmatis, M. bovis BCG	Temperate	No
Bo4	Siphoviridae	G	Unknown	M.smegmatis, M. tuberculosis	Lytic	Yes
VA6	Siphoviridae	–	–	M.smegmatis, M. avium intracellular	–	Yes
Mitti	Siphoviridae	K/K4	Soil	scrofulaceum M.smegmatis, M. tuberculosis	Temperate	Yes
Pomar16	Siphoviridae	A/A2	Soil	M. smegmatis	Temperate	Yes
AN9	Siphoviridae	–	–	M. smegmatis, M. avium-intracellulare-scrofulaceum complex	–	Yes

The family names (Siphoviridae, etc.) refer to historical morphological classifications. Under the current ICTV taxonomy, all these phages belong to the class Caudoviricetes.

### 1.5 Defense and counter-defense mechanisms in mycobacterium–phage interactions

**Figure 2:**
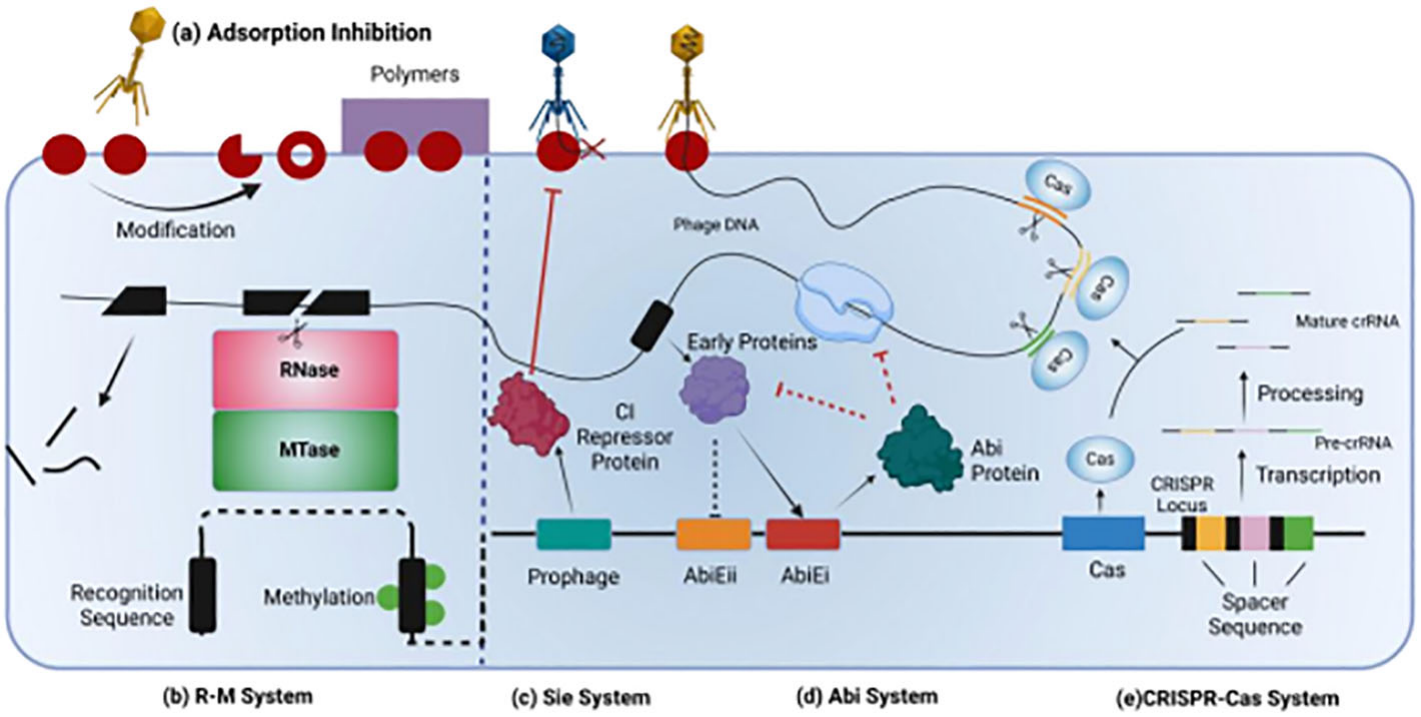
Anti-phage defense mechanisms in mycobacteria. (Created in https://BioRender.com).

Throughout long-term evolutionary pressure, mycobacteria have elaborated sophisticated, hierarchically organized defense systems comprising multiple functional modules that constrain phage attachment, penetration, propagation and dissemination. In response, bacteriophages perpetually refine countervailing strategies through genomic mutations, functional gene acquisition and regulatory network remodeling. This reciprocal adaptation drives a perpetual and dynamic co-evolutionary interplay between the host and the virus (Hampton et al., 2020; Hatfull, 2018). The major defense mechanisms employed by mycobacteria to circumvent phage-mediated lysis are illustrated in **Figure 2**.

Primarily, interference with phage attachment constitutes the most immediate defensive strategy employed by mycobacteria, typically encompassing cell wall composition modifications and surface receptors occlusion. Studies have shown that lipid components of the mycobacterial cell envelope play a pivotal role in adsorption efficiency. In *M. abscessus*, colony morphology correlates intimately with phage susceptibility. Smooth variants, characterized by the presence of glycopeptidolipids (GPLs) on the cell surface, tend to exhibit resistance to infection, whereas rough variants lacking GPLs due to mutations or differential expression of genes such as mps1 and mps2, are generally more susceptible. In addition, trehalose polyphleates (TPPs) have been identified as obligate co-receptors for mycobacteriophages *BPs* and *Muddy* infection, with TPP biosynthetic genes lesions abrogating phage binding and conferring tolerance. Furthermore, inactivation of the *lsr2* gene in *M. smegmatis* has been identified as a new defense mechanism. Lsr2 is a histone-like nucleoid-structuring(H- NS) negative regulator, which normally silences lipooligosaccharide (LOS) gene islandsand. Its deletion leads to derepresses these loci, causing the aberrant accumulation of phosphatidylinositol mannosides (PIMs) and consequent cell wall lipids remodeling that ultimately blocks phage *K4JX5* receptor engagement (Kołodziej et al., 2021). Moreover, specific genetic mutations have also been implicated in phage resistance. For example, the membrane-associated DNA exonuclease Mpr, when overexpressed or functionally altered, confers resistance to certain phages, potentially through DNA modification or stress pathway activation culminating in cell surface architectural alterations (Wang et al., 2025b).

When phages successfully adsorb and attempt to inject their genetic material into the host, mycobacteria deploy intracellular defense responses. Prophage-mediated heterotypic immunity represents one of the formidable barriers against phage invasion. Lysogenic prophages safeguard host from secondary infections through superinfection exclusion or homoimmunity. Proteins encoded by genes 30 and 31 of phage *Sbash* can specifically antagonizing cluster L phage entry, functionally augmenting canonical repressor-mediated homotypic immunity, thereby broadening the host defense spectrum (Dedrick et al., 2017a). Notably, the Sbash-encoded defense system specifically targets the phylogenetically distant phage Crossroads infections, such prophage-conferred resistance is particularly prevalent among *M. abscessus* clinical isolates and is considered a major contributor to inter-strain variability in phage susceptibility (Dedrick et al., 2017a). Indeed, approximately 75% of *M. abscessus* clinical isolates carry 1-8prophages encoding polymorphic toxin–immunity systems that can confer broad resistance against phages from multiple clusters (Sassi et al, 2014).

In addition to prophage-mediated mechanisms, mycobacteria utilize restriction– modification (R–M) systems to limit the foreign phage DNA invasion. These systems function by methylating host DNA by methyltransferases, thereby shields the host genome from cognate restriction endonucleases, while unmethylated exogenous phage DNA is recognized and degraded by restriction endonucleases. For example of *M. smegmatis*, the MsmAI methyltransferase modifies DNA at GATC sites, ensuring genomic integrity while targeting unmethylated viral genomes. Moreover, *M. tuberculosis* retains a CRISPR-Cas mediated adaptive immune system. The type III-A CRISPR-Cas system in *M. tuberculosis* can acquire fragments of invading phage DNA and integrate them into CRISPR arrays as spacer sequences. These spacers are transcribed into crRNAs, which guide Cas-mediated cleavage of homologous invading phage genomes, thereby conferring sequence-specific, heritable immunity (Rahman et al., 2022; Yeh et al., 2021).

Beyond the aforementioned mechanisms, the abortive infection (Abi) system represents the terminal defensive layer for mycobacteria against phage infection, often referred to an “altruistic” cellular suicide mechanism that sacrifices infected cells for communal protection (Aframian and Eldar, 2023). When phages successfully bypasses barriers of adsorption inhibition and DNA degradation, infected host cells can sense the aberrant viral activity, triggering the activation of host-encoded Abi proteins. Abi proteins suppress phage DNA replication or protein synthesis, ultimately inducing programmed cell death, thereby terminating phage propagation and safeguarding neighboring bacterial population (Aframian and Eldar, 2023). Recent studies have revealed that deletion of the *lsr2* gene in *M. smegmatis* not only blocks adsorption but can also trigger an uncharacterized abortive pathway under high MOI challenge by non- receptor-independent phage *F1GX13*, triggering rapid early-stage host cell death that effectively abortsphage propagation (Dedrick et al., 2017a). Abi proteins in *M. tuberculosis* similarly recognize phage DNA polymerase activity to execute suicide, halting the spread of infection. Collectively, these temporally coordinated defense mechanisms forms a multi-layered defensive front that enables mycobacteria to effectively resist phage invasion.

**Figure 3:**
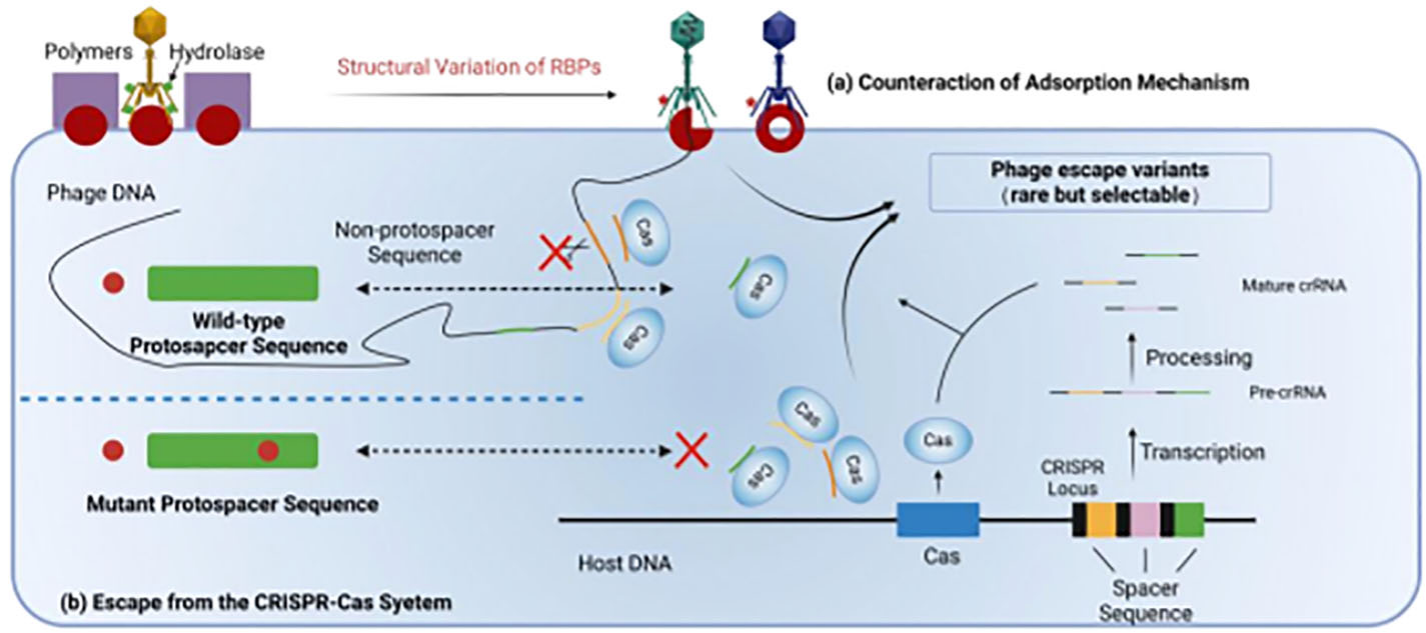
Phage counter-defense strategies in mycobacteria. (Created in https://BioRender.com).

Confronted with escalating host defensive architectures, mycobacteriophages have evolved multifaceted countervailing strategies through genomic variation, horizontal gene acquisition, and regulatory networks reprogramming (**Figure 3**). Some temperate phages can integrate directly into CRISPR-Cas loci, thereby disrupting immune surveillance. Others encode anti-CRISPR (Acr) proteins that inhibit Cas nuclease activity (Marino et al., 2020). The Acr protein encoded by phage *TM4* binds to Cas proteins and physically suppresses Cas nuclease activity, abrogating CRISPR-Cas- mediated recognition and cleavage. Lytic phages, in contrast, can achieve immune escape by accelerating lytic kinetics, compressing infection cycles to ~45 minutes to outpace host defense activation. In addition, the tail structural proteins, which dictate host adsorption specificity, frequently exhibit heightened mutation rates to circumvent host receptor recognition. Recent studies have further revealed that *TM4* subverts host immunity by hijacking the host eukaryotic-like serine/threonine protein kinase StpK7, leading to phosphorylation of the host transcription factor MSMEG_ 1198 and transcriptionally silencing BREX-like defense islands, effectively “silencing” host surveillance (Li et al., 2023). Investigation of *M. smegmatis* defense has also highlighted the central role of the lsr2 gene. Inactivation of lsr2 derepresses inactivation lipooligosaccharide (LOS) biosynthetic gene island. In response, receptor-dependent phage *K4JX5* can overcome this barrier through specific mutations in its tail fiber genes gp12/gp14 (Dulberger et al., 2023). Similarly, phage *L1HA1* can escape lsr2- deficiency-mediated immunity via gp30 tail protein mutation, thereby restoring infectivity toward lsr2-deficient strains. Intriguingly, *L1HA1* also encodes an Lsr2 homolog protein (PLP), which can mimic host regulatory proteins and restore bacterial susceptibility to phage. These evolutionary adaptations provide valuable natural templates for synthetic phage engineering and expand the mechanistic repertoire for phage-based antimycobacterial strategies.

These hierarchically organized resistance mechanisms between mycobacteriophages and their hosts collectively form a highly complex defense- counter-defense network. This intricate interplay also presents significant challenges for clinical application of phage therapy, necessitating strategies such as phage cocktail formulations or precision genome engineering to overcome host resistance and enhance therapeutic efficacy (Dedrick et al., 2019).

## 2 Applications of bacteriophages

The genus Mycobacterium comprises several important pathogens of profound public health significance, including *M. tuberculosis*, *M. bovis*, and *M. avium subsp. paratuberculosis* (MAP). These pathogens sustain themselves through persistent ecological cycles within soil, water, and dairy reservoirs. This environmental persistence underscores mycobacterial infections as a typical example of cross-species and cross-environmental transmission (Mirzayev et al., 2021). The escalating prevalence of multidrug-resistant (MDR) and extensively drug-resistant (XDR) mycobacterium strains has further complicated treatment, leading to prolonged therapeutic regimens, increased toxicity, and rising costs of disease control.

From the perspective of One Health, bacteriophage-based interventions offer a multifaceted strategy to dismantle mycobacterial transmission cascades and curtail antimicrobial resistance propagation across hierarchical scales. This can be achieved by encompassing pathogen suppression at zoonotic sources, biovigilance throughout the food production chain, and reducing persistent antimicrobial pressure within the environment.

### 2.1 Applications of mycobacteriophages in human health

Tuberculosis (TB), caused by *M. tuberculosis*, remains one of the most critical chronic infectious diseases globally (GBD 2019 Diseases and Injuries Collaborators., 2020). The emergence of multidrug-resistant tuberculosis (MDR-TB), together with TB/HIV co-infection, has posed substantial challenges to global TB control efforts (Ge and Wang, 2024). In recent years, global incidence of non-tuberculous mycobacterial (NTM) pulmonary disease has demonstrated sustained escalation (Ratnatunga et al., 2020; Wagner and Young, 2004),with the *M. avium complex* (MAC), *M. kansasii*, and *M. abscessus* constituting the predominant etiological agents (Johansen et al., 2020). Therefore, within epidemiological contexts where antimicrobial resistance and transmission dynamics potentiate one another, early mycobacterial detection, drug resistance profiling, and therapeutic innovation are essential for achieving durable disease control.

Bacteriophages depend on viable bacteria for infection, they produce plaques or lytic signatures upon successful infection and exhibit high host specificity. Together, these features provide a basis for the rapid detection and drug susceptibility testing of mycobacteria (Hatfull, 2018). Among them, the broad-host-range lytic phage *D29* has been widely used, partly because it remains stable under acidic and hypoxic conditions, making it suitable for rapid detecting latent *Mycobacterium tuberculosis*. Based on this, engineered reporter phages have been developed by introducing fluorescent protein or luciferase cassettes into the genomes of phages such as *TM4*, *D29*, *phAE159*, and *Φ²GFP10*, yielding quantifiable bioluminescent outputs. Upon productive infection of viable mycobacterial cells, these reporter phages facilitate expedited, high-sensitivity diagnostics as well as phenotypic drug susceptibility testing (Carrière et al., 1997; Jain et al., 2020; Liu et al., 2020; Rajagopalan et al., 2025). Furthermore, a phage amplification assay (PAA) monitors changes in phage replication under antibiotic pressure and can compress multidrug resistance TB screening timelines to mere days, enabling concurrent pathogen detection and resistance determination (Beinhauerova & Slana, 2021a).

Bacteriophages or receptor-binding proteins (RBPs) functionalization of electrochemical and surface plasmon resonance (SPR) transduction interfaces enables biosensor to capture subtle signal changes upon mycobacterial binding, obviating requisite phage replication kinetics and achieving minute-scale temporal resolution (Abril et al., 2022). For instance, bioluminescent sensors derived from the *SWU1* phage enable the rapid enrichment and detection of trace mycobacterial burdens in clinical samples. Beyond whole phages, phage-derived endolysins and cognate binding proteins have been engineered as immunocapture or enrichment tools to augment mycobacterial recovery efficiency from complex specimens (Abril et al., 2022; Aitken et al., 2025; Choi et al., 2023). These biosensors can also be combined with microfluidic chips, electrochemical detection, or isothermal amplification platforms to build compact and automated diagnostic systems. Such systems are particularly suited for resource-limited settings and primary healthcare facilities, as they reduce conventional culture-based diagnosis from days to hours (Abril et al., 2022; Shield et al., 2024).

Within the One Health framework, mycobacteriophages are no longer used only as an alternative to antibiotics. They are now being explored as part of more integrated treatment strategies encompassing prophylactic source containment, targeted delivery optimization, and synergistic bactericidal potentiation (Druszczynska et al., 2025). Precision engineering has expanded the transformative advances in phage therapeutic capability, including the deletion of lysogenic modules to prevent horizontal gene transfer, the modification of tail fibers to expand host range, and the integration of CRISPR-Cas systems for sequence-specific resistance gene ablation. One example is the phage *BPs*, which was engineered using BRED-mediated deletion of repressor gene 33. The resulting phage, *BPsΔ*33, has demonstrated promising safety and clinical efficacy in compassionate personalized phage cocktail therapy for disseminated *M. abscessus* infection, establishing critical proof-of-concept for subsequent large-scale, randomized controlled clinical trials (Dedrick et al., 2019; Druszczynska et al., 2025). Currently, the co-administration of phages with antimycobacterial drugs is emerging as investigational therapeutics for MDR-TB, XDR-TB, and recalcitrant NTM infections (Dedrick et al., 2023), with treatment algorithms increasingly guided by integrated drug susceptibility and phage lysis profiles of the isolated strains (Anastassopoulou et al., 2024). Prospective validation through multicenter clinical trials may ultimately elevate phage-augmented therapy from individualized salvage interventions to standardized adjunctive treatment for drug-resistant mycobacterial infections (Druszczynska et al., 2025).

### 2.2 Applications of mycobacteriophages in animal health

Amid intensifying global antimicrobial resistance (AMR), zoonotic bacterial infections pose escalating threats to agricultural sustainability and public health infrastructure. The main diseases of concern include bovine tuberculosis (bTB) caused by *M. bovis* and paratuberculosis (Johne’s disease) caused by *Mycobacterium avium* subsp. *Paratuberculosis* (*MAP*). These mycobacterial diseases share three features, including zoonotic transmissibility, environmental persistence, and the propensity for disseminating antimicrobial resistance.

Phage-based methods have been applied for identification and quantification of viable pathogens in milk. Commercial systems such as Actiphage^®^ and other optimized phage amplification methods can sensitively detect and enumeration of viable *MAP* in fresh sheep or bovine milk. These methods employ phage-assisted detection of milk and milk filter samples and significantly compress the diagnostic timeline for *M. bovis* from several weeks of culture to 24–48 hours. More recently, Actiphage Rapid technology has further reduced the detection time to less than 6 hours by using bacteriophages as efficient DNA lysis reagents combined with recombinase polymerase amplification (RPA), achieving detection sensitivity of up to 95% in bovine tuberculosis-positive herds (Beinhauerova & Slana, 2021b; Jones et al., 2020). Based on this, the Phage-*LAMP* platform further simplifies the detection process by integrating phage-mediated lysis with Loop-mediated Isothermal Amplification. This platform directly utilizes intracellular genomic DNA released by phages as templates for exponential signal amplification within 1 hour. Experimental evidence demonstrates that Phage-*LAMP* achieves a detection limit below 10 cells/mL for *MTBC*, *M. bovis*, and *MAP*, without requiring expensive thermal cycling equipment, substantially enhancing the potential for field-deployable rapid detection in resource-limited Settings (Shield et al., 2024). Comparative studies on bulk milk tanks (BMT) and blood samples highlight the phage amplification methods far surpass conventional culture in MAP detection capability, with phage-based detection achieving 22% positivity compared to merely 0.9% by traditional culture methods (Dane et al., 2026). Overall, phage amplification techniques resolve interference issues from complex samples on detection sensitivity, establishing rapid and specific diagnostic technologies capable of distinguishing viable from non-viable bacteria in surveillance and infection control.

### 2.3 Applications of mycobacteriophages in environmental control and ecological surveillance

Environmental compartments function not just as passive backgrounds but serve as important reservoirs sustaining mycobacterial persistence and enabling transboundary dissemination. Constructing surveillance architectures for viable pathogen detection and risk assessment across livestock systems, food production networks, aquatic/wastewater matrices and occupational environments represents a critical nexus for disrupting transmission cascades. In this context, mycobacteriophage- based technology offers unique advantages in environmental health assessment due to its ability to specifically identify viable bacteria with replicative capacity.

farm environments become contaminated through mycobacterial shedding via feces, milk, and respiratory secretions from infected livestock, establishing soil, bedding, water systems, and bioaerosols as persistent environmental reservoirs (Eisenberg et al., 2012). Slow-growing species, particularly *M. bovis* and *MAP*, can persist in these environments to form long-term pathogen reservoirs. Conventional culture and molecular detection methods face significant challenges in routine surveillance due to sample matrix complexity, high background microbial loads, and low target organism abundance (Pickrodt et al., 2023). Hybrid methodologies integrating magnetic bead capture with phage-mediated lysis demonstrate robust concordance for large-scale environmental monitoring, particularly for evaluating viable mycobacterial persistence through wastewater treatment processes and quantifying dynamic contamination in livestock waste systems (Bayat et al., 2021; Shivaram et al., 2023). In addition, phage-based pre-enrichment platforms such as Actiphage Rapid, coupled with downstream molecular detection, can significantly improve the detection of low- burden mycobacteria, enabling preemptive outbreak mitigation through targeted environmental interventions (Foddai and Grant, 2020).

*M. bovis* and *MAP* can infiltrate dairy processing systems via contaminated raw milk. Extensive food safety surveillance has documented viable *MAP* in retail milk, powdered formulations, and cheese, highlighting its long-term persistence within the food supply chain and associated consumer exposure hazards. Applying bacteriophage intervention during the pre-slaughter or rearing stages can significantly reduce the pathogen burdens entering downstream food processing, effecting source-level mycobacterial containment (Ferriol-González and Domingo-Calap, 2021; Goodridge and Bisha, 2011). For instance, phage cocktails have demonstrated to eliminate *M. smegmatis* in milk matrices within 24 hours with no bacterial regrowth (Ekiz et al., 2023), establishing innovative paradigms for controlling mycobacterial contamination in food processing. Compared to conventional chemical disinfection or antibiotic treatments, bacteriophages-mediated self-amplification within biofilms coupled with sustained endolysin deployment confers distinctive efficacy for biofilm management on food contact surfaces (Moye et al., 2018; Sillankorva et al., 2012).

Several settings represent pivotal for occupational exposure to mycobacterium. These include healthcare facilities, tuberculosis isolation wards, clinical microbiology laboratories, livestock farms, and slaughterhouses. Healthcare personnel demonstrate significantly elevated risk of tuberculosis compared to the general population (Daniel and Field, 2001; Matuka et al., 2021). Beyond clinical settings, veterinary professionals, farm laborers, and slaughterhouse workers occupy comparably hazardous occupational niches, with zoonotic transmission potential particularly accentuated in agrarian regions. In these settings, activities including animal restraint, manure system sanitation, and high-pressure spray washing can generate inhalable aerosols containing mycobacteria, increasing the risk of airborne exposure. The successful application of phage-based detection platforms across water, wastewater, and food samples provides both technical and conceptual support for extending phage technology to the monitoring of viable mycobacterial loads in occupational exposure environments (Yalan Gan et al., 2025; Nikolich and Filippov, 2020; Olea-Popelka et al., 2017). Incorporation of phage viable-pathogen monitoring architectures into occupational health frameworks may catalyze a paradigm shift from reactive individual case detection to proactive pre-exposure environmental risk characterization.

In intensive livestock production systems, antibiotic consumption the animal husbandry sector in some countries and regions accounts for more than 70% of total consumption. Such intensive application significantly accelerates the selection and amplification resistant bacteria within animal populations and contributes to cross- boundary dissemination of resistance genes through manure discharge, environmental contamination, and food chain transmission (Carroll et al., 2015; Van Boeckel et al., 2015). Consequently, mycobacteriophages can be considered as antibiotic alternatives or complements to conventional antibiotic strategies. By disrupting the cell wall of mycobacteria and degrading the biofilm matrix, phages can restore pathogen sensitivity to conventional drugs, thereby maintaining therapeutic efficacy while reducing clinical antimicrobial dosing concentration and frequency. Importantly, the implementation of phage-based prevention and therapeutic strategies at the production level can reduce pathogen burdens at their source, directly decreasing overall antimicrobial consumption (Gigante and Atterbury, 2019). This approach not only facilitates control of antimicrobial resistance within livestock systems but also effectively mitigates risks of resistant bacteria or resistance genes disseminating to human populations via environmental pathways or the food chain, thereby contributing to long-term ecological and public health security.

In the One Health framework, the application of mycobacteriophages has evolved beyond siloed, host-specific applications toward integrated, multi-domain deployment spanning, including human health, animal health, and environmental stewardship. The transformative potential of mycobacteriophages not only serves as alternatives to antibiotics for drug-resistant infections, but also lies in supporting transboundary risk governance and ecological modulation. Implementation of viability-based surveillance networks across healthcare settings, livestock systems, and water environments, combined with precision intervention frameworks, it may be possible to catalyze fundamental transition from reactive therapeutics to predictive alerting and upstream mitigation (Yehl et al., 2019). With the continued integration of synthetic biology, systems epidemiology, and environmental microbiomics, bacteriophages have the potential to emerge as precision bioregulatory tools operating seamlessly across the human-animal-environment interface, furnishing innovative strategies for sustainable, long-term control of mycobacterial disease.
